# Opportunity Assessment for Sustainable Aviation Fuel Production from Woody Biomass via Ex Situ Catalytic Fast Pyrolysis and Refinery Hydroprocessing

**DOI:** 10.1021/acs.energyfuels.5c03405

**Published:** 2025-11-05

**Authors:** Nicholas A. Carlson, Michael S. Talmadge, Michael B. Griffin, Abhijit Dutta, Kristiina Iisa

**Affiliations:** † 53405 National Renewable Energy Laboratory, 15013 Denver West Parkway, Golden 80401 Colorado, United States

## Abstract

Biofuels have become a promising solution to reduce emissions in hard-to-electrify transportation sectors, such as aviation. However, several biofuel conversion pathways will likely be needed to scale sustainable aviation fuel (SAF) production to meet lower carbon intensity goals when biomass feedstock availability constraints are considered. This study evaluates the future potential of the catalytic fast pyrolysis (CFP) pathway to contribute to U.S. SAF production goals as outlined in the U.S. Department of Energy’s (DOE) SAF Grand Challenge, understanding that the CFP pathway is still under development toward maturity and scaleup. National forestland resource data from the DOE’s 2023 Billion Ton Study are integrated with recent experimental results demonstrating end-to-end woody biomass conversion to SAF via CFP and hydroprocessing in a novel bioeconomy optimization framework. U.S. refinery hydroprocessing capacity is also considered with repurposing and coprocessing strategies. Results indicate that the CFP process can contribute up to 4.6 billion gallons of SAF annually by 2040, meeting 13% of the 2050 SAF Grand Challenge target while reducing the carbon intensity (CI) of the U.S. jet fuel pool by 16%. The study further explores optimal processing strategies, suggesting that colocating CFP operations with existing petroleum refineries and repurposing some U.S. hydrocracking capacity may offer significant cost advantages and enhance the commercial viability of the CFP pathway. These results underscore the CFP pathway’s potential to support the global aviation industry’s lower carbon intensity objectives while producing other renewable fuels and products.

## Graphical abstract

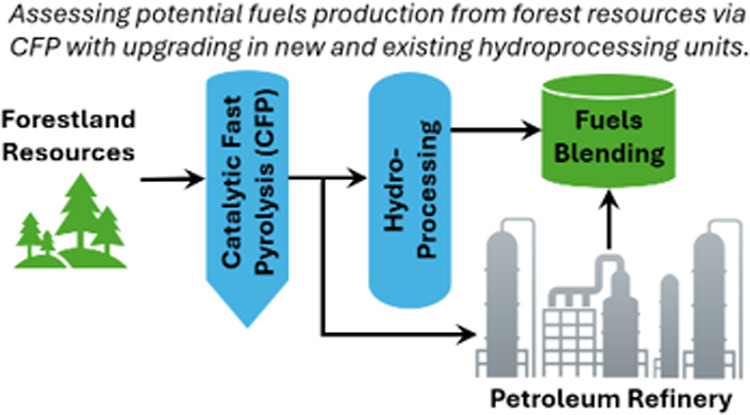

## 1 Introduction

Within the context of global initiatives to decrease carbon intensity (CI), biofuels have emerged as a leading candidate toward supplying transportation sectors that are difficult to electrify. This is particularly evident in the air transportation sector, which is being guided toward a net-zero economy by several initiatives that highlight the role of biofuels. Examples include the International Civil Aviation Organization’s (ICAO) Carbon Offsetting and Reduction Scheme for International Aviation (CORSIA), which has voluntary participation from 127 countries, the Science Based Targets initiative (SBTi), which has ambitious CI reduction commitments from 25 major airlines as of 2023, and the U.S. Department of Energy’s (DOE) Sustainable Aviation Fuel (SAF) Grand Challenge, which is prompting research and investment to meet volumetric targets of 3 billion (B)-Gal of SAF in 2030 and 35 B-Gal in 2050, with a minimum carbon intensity (CI) reduction of 50%.^1–3^

To meet these goals, several biomass and waste-to-SAF conversion pathways have been made eligible through ASTM certification with blend levels up to 50 Vol % with fossil-jet blendstocks.^4^ However, current production, estimated at 15.8 million gallons, or less than 0.1% of total jet fuel demand, primarily relies on fats, oils, and greases (FOG), as feedstock which have a limited availability of 4 million Dry-Tons/Yr, or approximately 1 B-Gal/Yr of SAF.^5–7^ To address these feedstock supply constraints, alternative pathways that convert feedstocks with more availability are attracting research interest and investment. These include alcohol-to-jet (ATJ) pathways, which convert agricultural wastes and residues into alcohols and then jet fuel through oligomerization. The Fischer–Tropsch pathway, which features the gasification of more widely available woody feedstocks and municipal solid wastes and yields a synthesized paraffinic kerosene blendstock, is another option.^8^

Another promising pathway is the catalytic fast pyrolysis (CFP) of solid wastes, particularly woody biomass.^4^ Fast pyrolysis is a relatively mature technology but produces a high oxygen content bio-oil with low miscibility, high acidity, and has a low energy density.^9^ Catalytic upgrading yields a stabilized bio-oil that can be used directly as a fuel-oil substitute or can be hydroprocessed to generate finished fuel blendstocks, including SAF. Besides gaining access to more abundant woody feedstocks, thermochemical upgrading and hydroprocessing steps may be compatible with existing petrochemical refining infrastructure, which carries the potential to significantly reduce capital expenses with considerations for needed upgrades to refinery equipment. Ongoing experimental work has assessed several catalytic upgrading reactor configurations, including in situ and fixed/fluidized bed ex-situ reactors, along with techno-economic and life-cycle analyses (TEA/LCA).^10,11^ Although pyrolysis-based conversion routes from biomass to fuels are not currently approved ASTM SAF pathways, work is ongoing within ASTM to qualify pyrolysis-based conversion of biomass as an approved pathway for SAF production. Recent work has experimentally demonstrated an end-to-end woody biomass to the SAF process via CFP and hydroprocessing with comprehensive yield, carbon efficiency, and SAF property assessments along with LCA results showing an 85–92% reduction in greenhouse gas emissions compared to conventional jet fuel.^12^ However, there is still a need to quantify the CFP pathway’s potential contributions to renewable fuel demands, such as SAF, based on current feedstock availability and yield estimates. Moreover, there is a need to explore what operating strategies are most promising to streamline the commercial-scale deployment of the technology. For the purposes of this analysis, the jet fuel cut from CFP and subsequent hydroprocessing to finished hydrocarbon fuels are considered an eligible contributor to SAF products.

This work supports existing CFP pathway research by simulating the potential of the United States to convert woody biomass into SAF through the CFP pathway with a novel bioeconomy optimization framework. National forestland feedstock availability estimates provided in the DOE’s 2023 Billion Ton Study are coupled with recent experimental results to assess the CFP pathway’s potential contributions to the DOE’s SAF Grand Challenge in terms of volumetric SAF production and carbon intensity (CI) reduction.^6^ Additionally, several processing strategies including standalone and colocated configurations are simulated in parallel, thereby allowing the optimization framework to identify the best way to leverage the technology with refinery hydroprocessing capacity considered. Results show that the CFP pathway can convert highly available renewable/waste forestland biomass into meaningful quantities of SAF while producing other renewable fuels and products, with the potential to reduce CI in several economic sectors.

The analysis approach and results presented here represent the initial demonstrations of NREL’s bioeconomy optimization efforts. The team recognizes and addresses limitations with the current framework and presents future enhancements needed to enable comprehensive and realistic bioeconomy optimization scenarios.

## 2 Methods

### 2.1 Catalytic Fast Pyrolysis for SAF Production

The CFP process has been a popular area of research to convert biomass and some organic waste feedstocks into fuels, chemicals, and products for over a decade.^10,13–16^ Key research topics have included balancing upgrading costs with pyrolysis oil qualities, primarily in terms of oxygen content, to optimize hydroprocessing operations to finished fuels and using pyrolysis oils as biointermediates to be integrated within existing petroleum refineries.^17^ Although the CFP process can produce a full spectrum of transportation fuels (LPG-range, gasoline, jet, diesel, and fuel oil/marine) when followed by hydroprocessing and fractionation steps, recent studies have focused on maximizing SAF production to prioritize decarbonizing a particularly hard-to-electrify transportation sector.^12^

A study that developed a series of integrated experimental campaigns to convert woody biomass to SAF was used as the experimental basis to model the CFP-to-SAF pathway.^12^ The experiments were designed around ex situ catalytic upgrading in a fluidized bed reactor using a ZSM-5 catalyst to resemble the conceptual process design shown in Figure S1. Several pyrolysis oils with oxygen contents of 17, 20, and 22 wt % oxygen were hydrotreated at 300, 350, and 385 °C, respectively, and then fractionated into gasoline, jet, and diesel blendstocks, respectively. Pyrolysis/upgrading yields, hydrotreating yields, and the resulting SAF blendstock compositions and properties were measured. These data were used to model the following unit operations: (1) pyrolysis oil yield and oxygen content as a function of the biomass to catalyst ratio (Figure S2), (2) hydrotreating yields as a function of pyrolysis oil oxygen content and temperature (Figure 1), and (3) fractionation yields and SAF blending properties as a function of pyrolysis oil oxygen content and temperature (Table S1).

**Figure 1:**
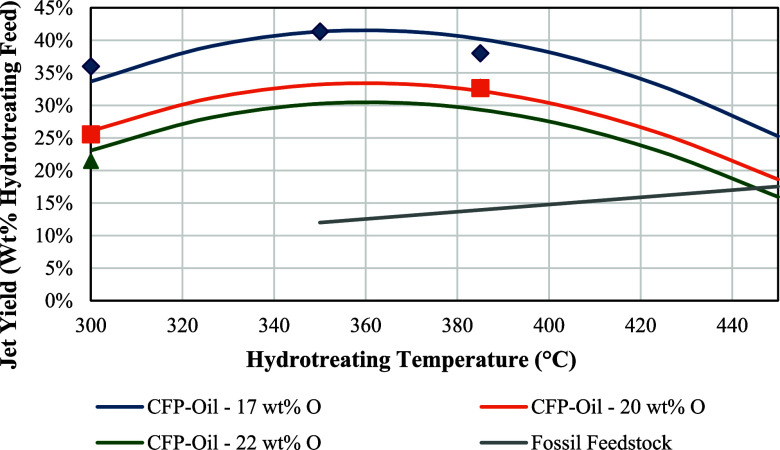
Jet blendstock yields after hydrotreating CFP-oils with 17, 20, and 22 wt % oxygen content at 300, 350, and 385 °C, respectively, with a NiMo/Al_2_O_3_ catalyst compared with petroleum hydrocracking yields sourced from an industry linear-programming (LP) optimization model.^18^

Techno-economic and life-cycle analyses (TEA/LCA), consistent with a series of comprehensive design reports, were also referenced for key economic and environmental metrics corresponding to a standalone version of the process.^11,12,19^

Petroleum colocation options including coprocessing and repurposing were also considered to identify preferred processing strategies. Simple capital cost reduction assumptions were applied relative to standalone biorefinery costs for hydroprocessing and utilities to represent refinery repurposing and coprocessing opportunities. Percentages of standalone hydroprocessing and utilities capital costs were assumed to be 50% and 25% for repurposing and coprocessing, respectively. These numbers were chosen to reasonably estimate turnaround and retrofitting costs that refiners might encounter when modifying units to process bio-oils. Also, a standalone CFP process without hydrotreating, where the final product is the upgraded catalytic pyrolysis bio-oil intermediate, was also considered. This allowed the model to decide whether transporting woody biomass, pyrolysis bio-oil, or finished jet blendstock was the most economical. All CFP processing strategies considered are shown graphically in Figure S3.

### 2.2 Feedstocks

The forestland resources reported in the 2023 Billion Ton Study were utilized to quantify biomass feedstock availability for the CFP-to-SAF pathway since the experimental results used to model SAF yields and properties used a 50% clean pine and 50% forest residues mixture.^12,20^ The study documents 88,971 individual feedstocks that can be sustainably harvested on an annual basis, categorized by types such as softwood, hardwood, mixed-wood, and logging residues, along with specific locations, prices, and scenario assignments. To simplify the analysis, specific locations were omitted, and feedstocks were grouped by price, which were rounded to nearest tens, resulting in four price categories of 40, 50, 60, and 70 $/Dry-Ton, for which resource availabilities were summed. Current forestland resources were assumed to fall in the $70 per dry ton category to align with Idaho National Laboratory’s (INL) Woody Feedstock 2020 State of Technology Report which was used in the TEA and LCA performed for the CFP-to-SAF pathway referenced in this study.^12,21^

The scenarios presented in the 2023 Billion Ton Study—current, near-term, mature-market low, mature-market medium, mature-market high, and emerging shown in Figure S3—were aligned with the years 2025, 2030, 2035, 2040, 2045, and 2050, respectively, spanning the analysis window relevant to the SAF Grand Challenge. The alignment of scenarios and years is directly justified for the current (2025) and near-term (2030) scenarios as specified in the 2023 Billion Ton Report.^6^ An assumption was made that the mature market low, medium, and high scenarios, characterized by increasing market pull for biomass, will unfold between years 2035 and 2045 and emerging resources will be available in 2050. The severity of this assumption is alleviated by the fact forestland resource growth stagnates by 2035, after which 63 million dry-tons per year can be sustainably harvested on top of the 143.7 million dry-tons already consumed.^20^ Finally, availabilities were linearly interpolated between each five-year increment and smoothed by averaging with the two nearest data points to provide yearly availability, as illustrated in Figure 2.

**Figure 2:**
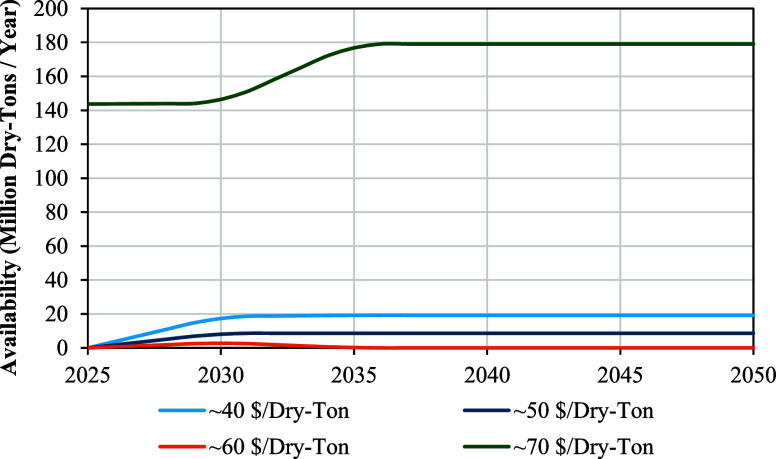
Annual forestland resource availabilities from the 2023 Billion Ton Study grouped together by rounded prices of 40, 50, 60, and 70 $/dry-ton with the following scenario/year alignments: current (2025), near term (2030), mature market low (2035), mature market medium (2040), mature market high (2045), and emerging (2050). Current forest feedstock consumption was assumed to be in the 70 $/dry-ton category.

Differences in feedstock price and carbon intensity pertaining to the standalone and refinery colocation configurations were also determined since transportation costs are an important consideration when processing low-density biomass. Petroleum refinery locations were sourced from the Energy Information Administration’s (EIA) Refinery Capacity Report.^22^ These locations were used to calculate straight-line distances between each forestland feedstock reported in the 2023 Billion Ton Study and each U.S. refinery with an operational hydrocracker. Minimum distances to a hydrocracker were determined for each feedstock, with the assumption that every unit has the ability and capacity to be retrofitted for CFP bio-oil hydroprocessing. Availability-weighted averages of the minimum distances were then calculated for each feedstock price category to estimate national averages of the transportation distances between forestland resources and existing refinery hydrocrackers. Moreover, transportation costs and emissions of 0.051 $/Ton-Mile and 29 gCO2e/Ton-Mile were assumed to compute the price and CI of transported feedstocks arriving at the refinery gate, on top of the roadside price values reported in the Billion Ton Study and CIs used for detailed pathway LCA as shown in Table 1.^12,20^ As an aside, percentages of forestland resource total availability as a function of distance to a hydrocracker were calculated, as shown in Figure S4.

**Table 1:** Key Pricing and Carbon Intensity Assumptions around Standalone and Co-Located Facilities^12^

			price ($/MJ) | CI (gCO_2_e/MJ)	
feedstock	price ($/dry-ton)	average distance to hydrocracker (miles)	standalone	co-located
logging residues	40	204	0.0023 | 15.96	0.0046 | 17.42
other forest waste	50	199	0.0028 | 15.96	0.0051 | 17.42
forest processing wastes	60	300	0.0034 | 15.96	0.0057 | 17.42
current resources + small-diameter trees	70	223	0.0039 | 15.96	0.0062 | 17.42

### 2.3 Sustainable Aviation Fuel Product

The CFP-to-SAF product was blended with fossil blendstocks including straight-run, hydrotreated, and hydrocracked kerosene such that the final product would meet ASTM D1655-22 Jet A blending property specifications.^23^ Fossil blendstock and CFP-to-SAF blending properties are shown alongside ASTM specifications in Table S2 for reference. Jet production levels (encompassing SAF) were also forced to equal the jet fuel demand projections outlined in the baseline scenario presented in the Energy Information Administration’s (EIA) 2023 Annual Energy Outlook (AEO).^24^ Availabilities of each fossil blendstock were estimated by multiplying unit capacity values in the EIA’s 2023 Refinery Capacity Report by baseline kerosene yields used in a refinery optimization modeling framework that has been featured in other analyses.^18,25,26^

### 2.4 Bioeconomy Optimization Framework

A Python-based bioeconomy optimization model (BiOpt) developed at the National Renewable Energy Laboratory (NREL) was leveraged to simulate the CFP-to-Jet pathway operating at scale using U.S. forestland resources. The model is designed to optimize limited biomass resource distributions over time across one or multiple conversion pathways according to a user-defined objective function. The model’s development began with the aim of adopting a more comprehensive approach to TEA/LCA by considering multiple pathways simultaneously working toward common objectives and to address questions regarding optimal uses of biomass. While several frameworks already address the cross-sectoral and system-level implications of biofuel production, BiOpt’s primary intention is to preserve the rigor of detailed process models and corresponding TEA/LCA. Additionally, the model is designed to be easily updatable, allowing for the seamless incorporation and analysis of key technological advancements in biofuels research within the context of a broader bioeconomy. BiOpt’s underlying architecture is a network model composed of layers which correspond to different stages of a prospective bioeconomy converting biomass into intermediates and/or into finished fuels with relevant specifications as shown in Figure 3. The model’s primary components are discussed in further detail below.

**Figure 3:**
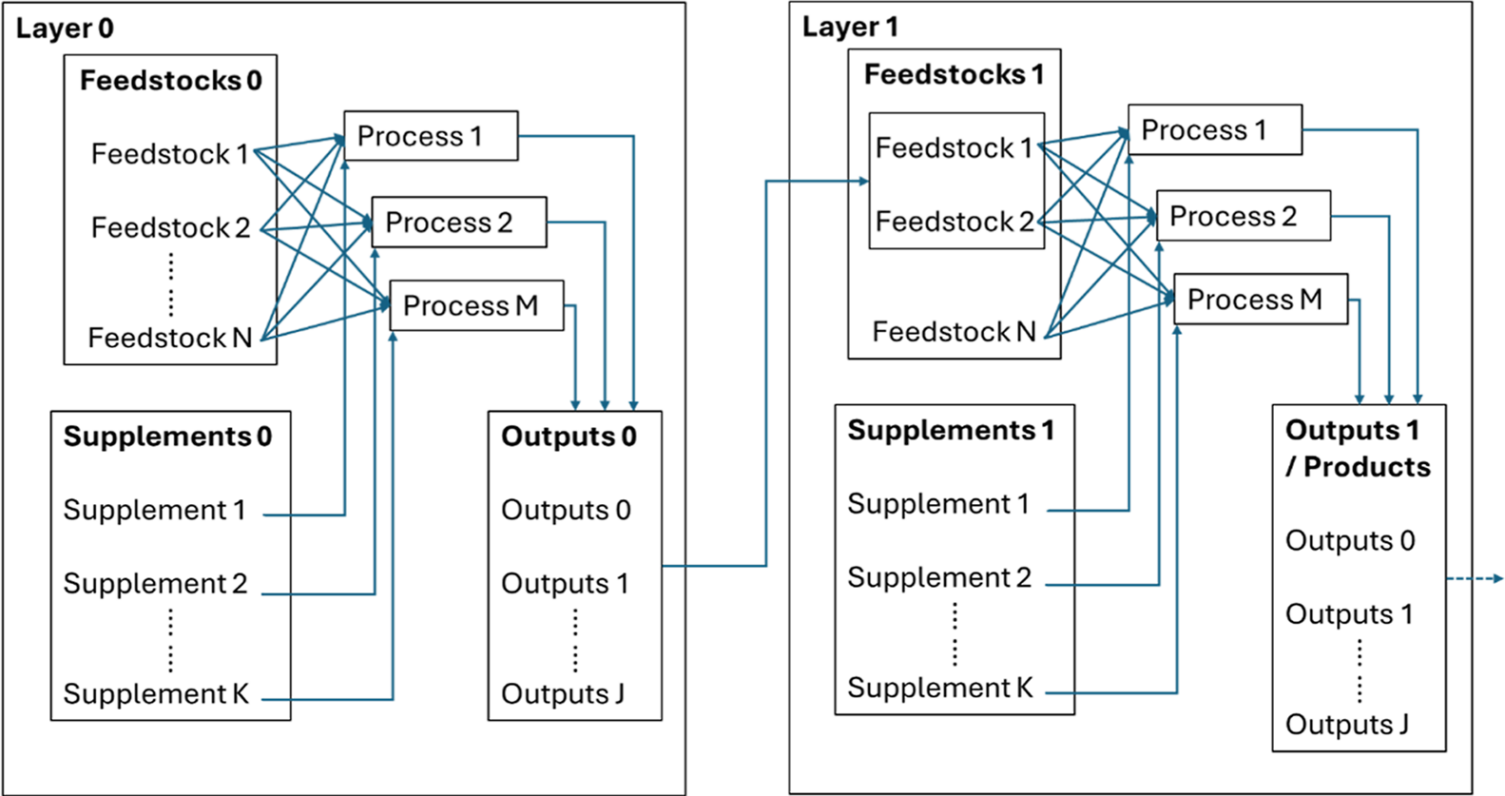
Model architecture of the bioeconomy optimization framework (BiOpt) used to simulate the CFP pathway’s scaled deployment. Model components include feedstocks, supplements (without properties), and outputs which are grouped into layers. The streams flow in and out of process meta-models which simulate detailed process models with TEA/LCA.

#### 2.4.1 Streams

Each layer is composed of feedstocks, supplements, and outputs, which are different categorizations of streams that are handled differently in the network model. Feedstocks have defined availabilities, locations, properties, and transportation costs associated with moving to separate processes in other layers. Their properties are blended on a mass or volume basis, as defined by the user, before entering a conversion pathway, which enables critical material attributes of the blended feed to be calculated and to influence process model calculations. They can also be passed directly to subsequent layers to facilitate configuration building.

Supplements, in contrast, do not have predefined availabilities or properties but are purchased as needed by each process. They are included in the framework to monitor/report resource consumption and to facilitate product carbon intensity tracking, as discussed below. Examples of commonly used supplements are electricity, natural gas, steam, and other utilities.

Outputs are similar to feedstocks and capture the quantities and properties of products calculated by each process model. Moreover, they can be passed to other layers and combined with other predefined feedstocks, which enables the model to capture dependencies and relationships between processes. In the final layer, outputs are blended to calculate finished fuel properties, which are then subjected to user-defined specifications in the form of inequality constraints. A materials database is used to fill in unspecified properties, prices, and carbon intensities with default values sourced from the literature and commercial databases such as Greenhouse gases, Regulated Emissions, and Energy use in Technologies (GREET).^27^

#### 2.4.2 Processes

NREL has a large library of rigorous process models developed in Aspen Plus that are continuously informed and updated by experimental research. From a process model, detailed TEA is conducted to determine the capital expenses (CAPEX), operating expenses (OPEX), and minimum fuel selling prices (MFSP) associated with each pathway. Additionally, process models inform life-cycle analyses (LCAs) to determine the emissions produced by each process design. The main challenge in integration of full process, TEA, and LCA models into an optimizable system is that they are (a) highly rigorous and take too long to execute and/or (b) conducted using different software packages that are difficult to integrate. Therefore, lower-order meta-models are developed from the rigorous modeling frameworks using automated case generation scripts and output data generated from rigorous models to represent each conversion pathway within BiOpt. The resulting meta-model is computationally inexpensive approximation of a full-order model and captures essential input–output behaviors of the complex model required for resource-to-conversion pathway optimizations. The BiOpt framework is designed to incorporate meta-models to represent conversion processes and dramatically speed up the optimization process. Figure 4 provides a graphical representation for the development of meta-models based on (a) an input matrix of critical variables required to appropriately represent each pathway, (b) automated case generation scripts integrated with rigorous process, TEA, and LCA models, and (c) output data matrix representing the meta-model basis. The result is a library of flexible python representations of each conversion process that considers feedstock quantities, feedstock properties, and select process parameters as inputs and calculates corresponding product yields, capital costs, operating costs, MFSPs, and emissions, which are assumed to be sufficient to summarize the process operation within the broader bioeconomy.

**Figure 4:**
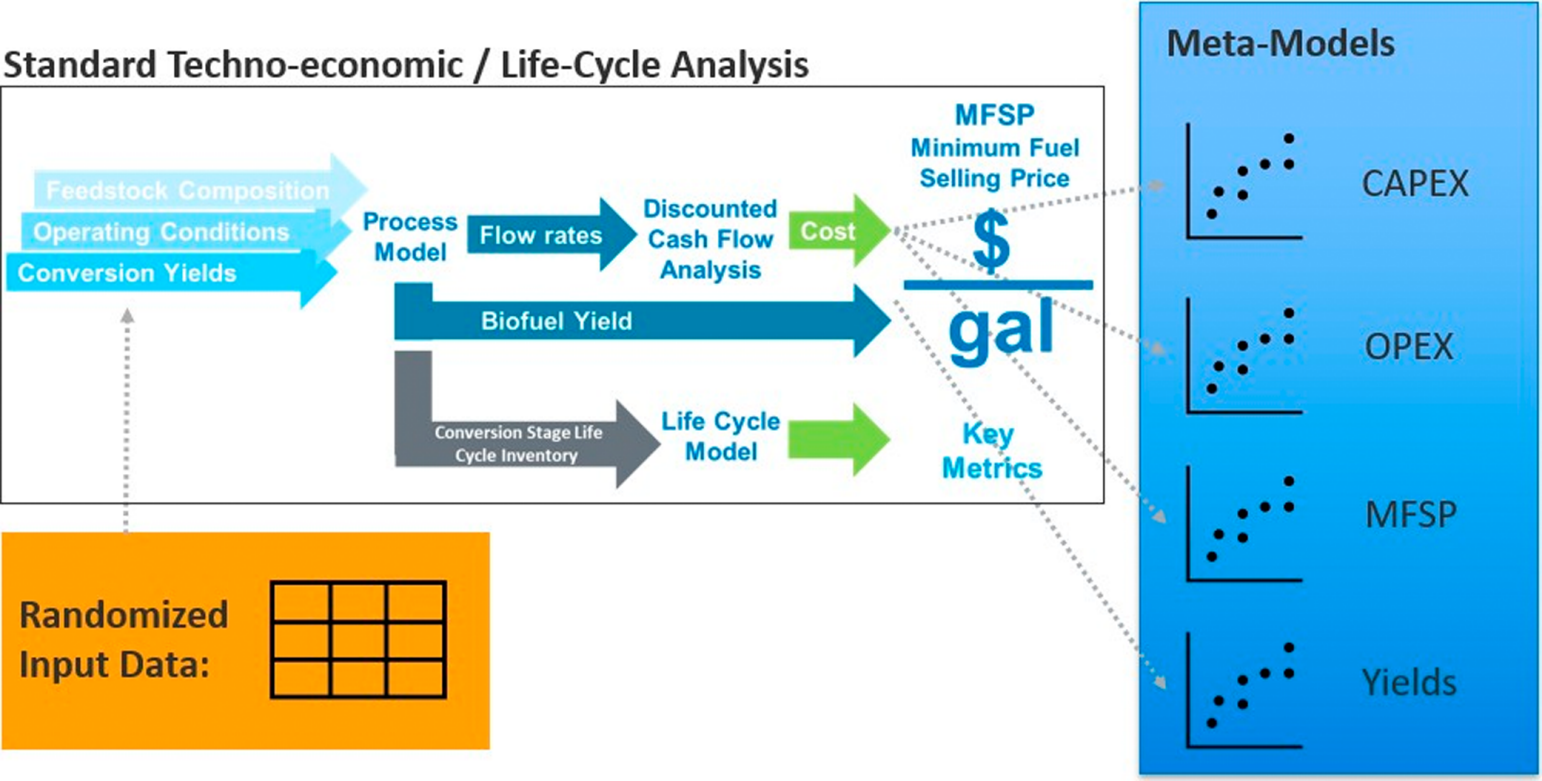
A graphical depiction of the meta-model development approach combining experimental research, process design, and life-cycle assessments into a single, lower-order model used to represent biofuel conversion pathways in BiOpt.

The meta-models can be configured in any logical fashion and calculated quickly, which makes them amenable to optimization and complex configurations. The meta-models can also be increased or decreased in rigor and complexity depending on the goals of each analysis effort.

#### 2.4.3 Carbon Intensity Tracking

The carbon intensities of each input stream in BiOpt are either manually input or default values can be automatically pulled from the materials database. After each process meta-model is calculated, the model multiplies each process input carbon intensity by its energy flow, sums the total emissions attributed to all process inputs, and then distributes that total across each process output according to their energy flows. In this way, the carbon intensities of each feedstock can flow through each bioeconomy process to the final products on an energy allocation basis. The procedure used to calculate the carbon intensities of process outputs, extending to finished products (i.e., SAF), is shown in the equation set below where inputs are both feedstocks and supplements.

1 $${GHG}_{ins}\left(\frac{g{CO}_{2}eq}{yr}\right)=\sum_{ins}{CI}_{in}\left(\frac{g{CO}_{2}eq}{MJ}\right)\cdot F_{in}\left(\frac{MJ}{yr}\right)$$

2 $$F_{outs}\left(\frac{MJ}{yr}\right)=\sum_{outs}F_{out}\left(\frac{MJ}{yr}\right)$$

3 $${GHG}_{out}\left(\frac{g{CO}_{2}eq}{yr}\right)={GHG}_{ins}\left(\frac{g{CO}_{2}eq}{yr}\right)\cdot \left(\frac{F_{out}}{F_{outs}}\right)$$

4 $${CI}_{out}\left(\frac{g{CO}_{2}eq}{MJ}\right)={GHG}_{out}\left(\frac{g{CO}_{2}eq}{yr}\right)/F_{out}\left(\frac{MJ}{yr}\right)$$

## 3 Results and Discussion

BiOpt was configured to estimate the pathway’s volumetric SAF production potential by forcing the model to distribute all available forestland feedstock across the various CFP process configurations while minimizing costs by maximizing the total system’s net-present value (NPV).

### 3.1 SAF Production Potential

Figure 5 shows the resulting volumetric SAF production alongside a quadratic interpolation of the SAF Grand Challenge volumetric targets of 3 and 35 B-Gal/Yr in 2030 and 2050 with the simplifying assumption of no SAF production in 2025. The EIA’s baseline jet fuel demand projection, which the model was forced to satisfy by blending the CFP-to-SAF blendstock with fossil kerosene blendstocks, is also shown for reference. Additionally, carbon intensities for fossil jet (89 gCO_2_e/MJ), the jet pool after blending CFP-to-SAF with fossil blendstocks, and the SAF Grand Challenge Target of a 50% reduction in greenhouse gas (GHG) emissions from the baseline (44.5 gCO2e/MJ) are also shown in the figure as dotted reference lines..

**Figure 5:**
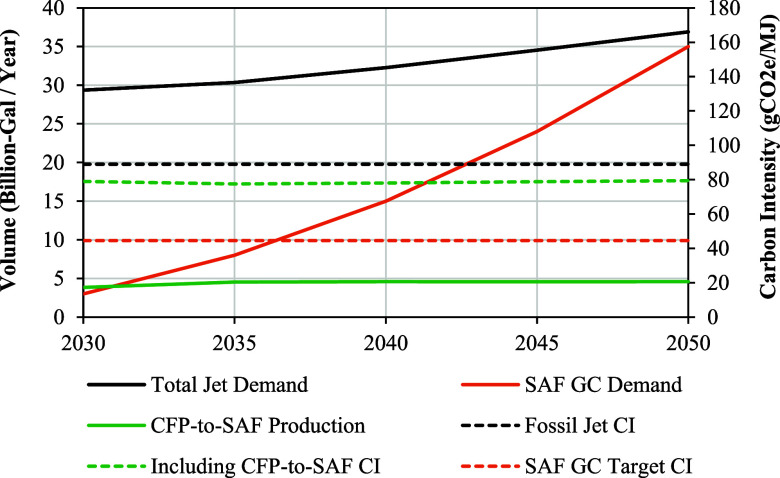
CFP-to-SAF volumetric production potential displayed alongside the EIA’s 2023 AEO baseline U.S. jet demand projection and a quadratic interpolation of the SAF Grand Challenge Targets. The jet pool carbon intensity after blending CFP-to-SAF is also shown next to the baseline fossil jet and the SAF Grand Challenge Goal of 50% reduction values.

Results show that the CFP-to-SAF pathway has the potential to convert all U.S. forestland feedstocks into 3.8 B-Gal/Yr on-spec SAF starting in 2030, which exceeds the 3 B-Gal/Yr intermediate goal stated in the SAF Grand Challenge. Additionally, volumetric production potential increases to 4.6 B-Gal/Yr by 2040 extending to 2050 as more forestland feedstock becomes available as shown in Figure 2. This volume equals a meaningful contribution of 13% of the 35 B-Gal/Yr targets in the SAF Grand Challenge by 2050. This contribution reduces the U.S. jet pool carbon intensity by 16% or 11% using either the SAF Grand Challenge volumetric target or the EIA’s U.S. jet demand projection of 37 B-Gal/Yr in 2050, respectively.

### 3.2 Co-Product Potential

Aside from SAF, the CFP pathway also produced meaningful quantities of other transportation fuels and renewable electricity, as shown in Figure 6. Finished fuel specifications were not imposed on fuels other than SAF because gasoline, diesel, and marine fraction blending property measurements were not taken in the experiments referenced herein. Therefore, these quantities should be viewed as blendstocks with the potential to meet finished fuel specifications when combined with other fossil and renewable blendstocks.

**Figure 6:**
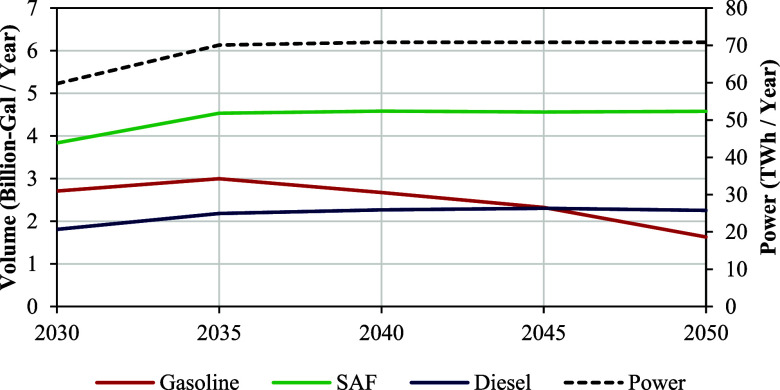
Gasoline/diesel fuel blendstock and renewable electricity coproduct quantities shown alongside SAF production from the CFP-to-SAF pathway utilizing all available U.S. forestland feedstocks.

The NREL CFP process design utilizes combustors to burn char/coke from the surfaces of catalysts and other fluid bed materials to generate heat and electricity for other process areas or export.^12^ If char combustion is forgone and electricity needs are met from other sources, the CFP pathway has the potential to generate a biochar coproduct which is being researched as soil amendment, animal feed supplement, building material additive, and other applications.^28–30^ Table 2 places biochar and other coproduct quantities into context as a percentage of market demand projections. Although SAF production has the potential to contribute the most in terms of market share, significant quantities of renewable gasoline and diesel blendstocks are also generated, thereby enabling the pathway to supply almost 5% of the US transportation fuel demand. Moreover, 2% of U.S. power could be supplied as a coproduct, which could be highly impactful in select regions of the U.S. with large forestland resources such as the Southeast.

**Table 2:** Maximum Co-Product Production Quantities Reported and Taken as a Percentage of Market Demand Estimates Sourced as Follows According to the Co-Product Type; (1) Fuels: EIA’s 2023 AEO Demand Estimates in 2050,^24^ (2) Power: U.S. Electricity Generation in 2023,^31^ (3) Biochar: Global Production in 2023 ^32^

product	CFP production potential	potential market share
gasoline	3.0 B-Gal/Yr	2.6 Vol %
SAF	4.6 B-Gal/Yr	12.3 Vol %
diesel	2.3 B-Gal/Yr	4.0 Vol %
total fuel	9.8 B-Gal/Yr	4.7 Vol %
power	70 TWh/Yr	2% of 2023 US Production
^⧧^biochar	25 million MT/Yr	8000 Wt % of Global Production in 2023

### 3.3 Processing Strategy

Several configurations of the CFP-to-SAF process were considered including standalone SAF production and standalone or colocated bio-oil intermediate production with transportation to a refinery hydrocracker for hydroprocessing. Additionally, coprocessing and repurposing options were made available to the refinery hydrocracker. Among these options, BiOpt exclusively selected the colocation and repurposing option, thereby signaling it as the most cost-effective configuration of the CFP-to-SAF pathway. This determination can be reduced to two decision points that differentiate the various process configurations.

First, hydroprocessing and utility capital cost reductions enabled by colocating pyrolysis unit operations with an existing refinery were sufficient to overcome the added expense of shipping low-density woody biomass instead of a denser bio-oil intermediate. This trade-off is heavily dependent on the simplified capital cost reduction assumptions made where costs were assumed to be 25% and 50% for coprocessing and repurposing, respectively. Therefore, minimum feasible selling prices (MFSPs) were calculated for standalone and colocated configurations with various capital cost percentage assumptions as functions of feedstock distance as shown in Figure 7A. From these results, another informative plot in Figure 7B presents the breakeven point between feedstock transport distance and the capital costs associated with repurposing refinery units for CFP upgrading. The figure shows that the maximum economically feasible feedstock transport distance is highly sensitive to the capital costs associated with refinery integration. If capital costs for repurposing refinery units are substantially lower than the capital costs for new, standalone hydroprocessing units, then refiners have opportunities to import feedstocks or CFP intermediates over much greater distances.

**Figure 7:**
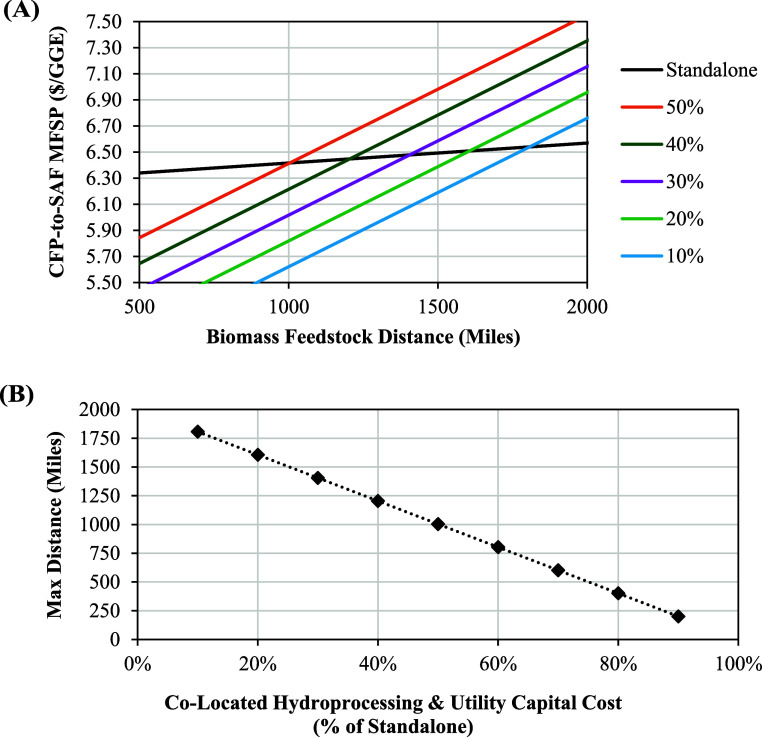
Sensitivity analysis results for (A) MFSP results for standalone and colocated biorefineries with various capital cost percentage assumptions as functions of feedstock distance and (B) breakeven feedstock distance as a function of the colocated capital cost percentage. Breakeven is where standalone MFSP equals colocated. Beyond maximum distance, colocated option has a cost advantage.

Based on Figure 7B and the average biomass feedstock distance of 230 miles calculated using the values reported in Table 1, hydroprocessing and utility capital costs could be 88% of standalone before standalone bio-oil production would be more economical than colocating pyrolysis operations. This result suggests that colocation could be preferable in most scenarios depending on regional biomass transportation and hydrocracker repurposing costs.

Second, repurposing was selected over coprocessing despite the strategy’s significantly higher capital costs. To understand the optimizer’s preference for repurposing, hydrocracking temperatures for the repurposed unit dedicated to bio-oil hydroprocessing and the refinery unit upgrading fossil feedstocks were compared as shown in Figure 8. Jet yields resulting from the two units are also displayed for reference.

**Figure 8:**
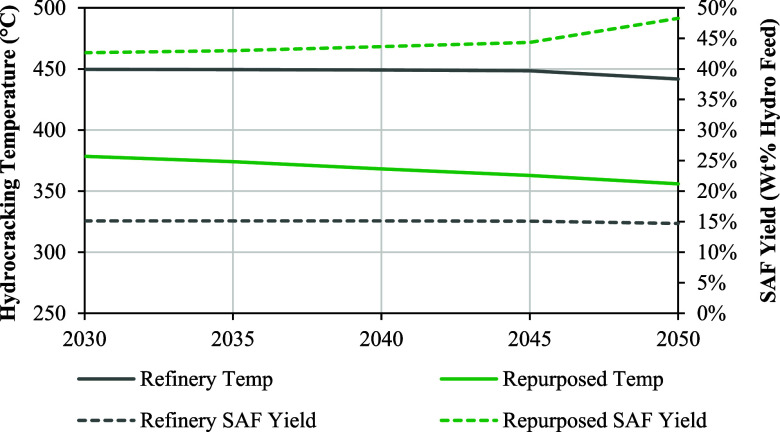
Optimal hydrocracking temperatures and resulting SAF yields (wt % of hydrocracker feed) for the refinery and repurposed for bio-oil hydrocrackers from years 2030 to 2050.

As shown in Figure 8, the optimal hydrocracking temperatures found for the refinery (450 °C) and repurposed (∼370 °C) units differed significantly. Noting that the only production constraint implemented in the model was for jet/SAF, this discrepancy can be explained by the SAF yield curves shown in Figure 1 where maximum jet yields are achieved at 450 and ∼370 °C for fossil and CFP bio-oil feedstocks, respectively, which align with the temperatures determined by the optimizer. Therefore, the optimizer determined that spending capital to repurpose some hydrocracking capacity (11.4 B-Gal/Yr or 30 vol % of U.S. capacity) was worthwhile to allow fossil and bio-oil hydroprocessing operations to be optimized independently to maximize jet production. This result suggests that aligning fossil and bio-oil hydroprocessing conditions could improve the economic viability of coprocessing which could further reduce capital costs and increase operational flexibility for refiners interested in processing low-carbon bio-oils. However, repurposing could come with the benefits of easier biocarbon tracking, life-cycle assessment, and determining eligibility for incentives.

### 3.4 Limitations of Study

While this study provides valuable insights into production strategies for CFP and potential product volumes, several areas for improvement are identified for future BiOpt model development and analysis. As NREL continues to develop resource-conversion optimization capabilities, the following represent the primary focus areas:

- Incorporate additional feedstocks and conversion pathways for more comprehensive analysis and development of economically and environmentally attractive roadmaps.
- Increase the geospatial resolution of (a) waste resource locations and available quantities and (b) existing refinery and fuel distribution infrastructure. This will enable (a) nonlinear optimizations of scale versus logistics costs and (b) facility siting optimizations where the model will identify the most promising locations for conversion technology deployment based on local resources. Geospatial optimizations would also lead to new capabilities like supply chain feasibility assessments (e.g., truck/rail delivery frequency). Correspondingly, increase detail in transportation cost calculations and add options including rail, marine, and pipeline transport.
- Incorporate realistic infrastructure constraints. Integration of CFP oil and other alternative intermediates with existing infrastructure will introduce new challenges and constraints that must be properly represented in modeling and analysis. For example, refinery integration via hydroprocessing will increase refinery demands for hydrogen, add complexity to reactor exotherm management, and increase wastewater production. Based on results of parallel experimental work, engineering studies, and industry engagement, NREL will incorporate limits, constraints, and costs associated with utilizing existing infrastructure.
- Enhance meta-models to increase property specificity of the feedstocks derived from waste resources and corresponding conversion impacts. This would allow for more accurate modeling of feedstock availability and conversion potential through increasing detail/order in meta-models. This may also include constraints, limitations, and consequences in the supply chain as a function of material quality for improved risk assessments. In these areas, NREL will seek to integrate experimental data, process/TEA models, and applications of artificial intelligence and machine learning (AI/ML) to inform/create enhanced meta-models.
- Incorporate a modeling basis for industrial learning to model the transition of first-of-kind deployment through technology maturity.
- Enable optimizations that consider alternative revenue streams such as federal, state, and local policy incentives.
- Refactor BiOpt as an open-source model. Refactoring the code will improve the code quality, enable simpler and faster development and maintenance, and allow for broader collaboration.
- Seek connections with other NREL modeling platforms. In addition to expanding the scope of biomass feedstocks and conversion pathways, goals for BiOpt development include linking the model to electrical grid models and databases for alternative feedstocks like CO_2_ and municipal solid waste.

## 4 Conclusions

This analysis suggests that the CFP pathway is capable of meaningfully contributing to SAF and other renewable fuel production goals when deployed at scale. The technology is advantaged by its list of high-availability candidate feedstocks, including the 207 million Dry-Mt/Yr of woody feedstocks considered herein and potentially 231 and 205 million Dry-Mt/Yr of municipal solid waste and agricultural residues with modified process designs. Additionally, hydroprocessing CFP bio-oils presents an opportunity for existing petroleum refineries to integrate low-carbon feedstocks while significantly reducing capital costs for SAF producers. Results suggest that colocating pyrolysis processes with refinery infrastructure could be a cost-effective strategy given average U.S. woody feedstock and refinery locations. Moreover, hydrocracker repurposing appears to be an effective way to upgrade CFP bio-oils if optimal fossil and bio-oil hydroprocessing conditions do not align in specific implementation scenarios. Key research areas that could further derisk the CFP to SAF pathway include improving SAF carbon yield, reducing feedstock costs, and reducing petroleum refinery retrofitting costs to process pyrolysis oils.

This work also represents a key achievement in combining resource data, experimental data from emerging conversion technologies, and a basis for the existing fuel production infrastructure to optimize utilization of bioeconomy resources. There are many opportunities to further develop and expand optimization capabilities and enhance them through emerging computer science.

## Supplementary Material



## Notes

Process flow diagram of the ex situ CFP process with hydroprocessing; CFP bio-oil yield and oxygen content as functions of the biomass-to-catalyst ratio in the pyrolysis process; CFP bio-oil hydroprocessing yields and SAF properties for various CFP bio-oil oxygen contents and reactor temperatures; graphical overview of CFP-based SAF production strategies utilized in the BiOpt model; biomass resource availabilities for each scenario presented in 2023 Billion Ton Study; percent of U.S. forestland feedstock in 2023 Billion Ton Study as a function of distance to a U.S. refinery hydrocracking unit; and ASTM Jet-A fuel specifications imposed in the BiOpt model (PDF)

## Abbreviations

AEO: Annual Energy Outlook
ASTM: American Society for Testing and Materials
ATJ: alcohol-to-jet
CAPEX: capital expenses
CFP: catalytic fast pyrolysis
CI: carbon intensity
CORSIA: Carbon Offsetting and Reduction Scheme for International Aviation
DOE: U.S. Department of Energy
EIA: Energy Information Administration
FOG: fats oils and greases
GHG: greenhouse gas
GREET: Greenhouse gases, Regulated Emissions, and Energy use in Technologies
ICAO: International Civil Aviation Organization
INL: Idaho National Laboratory
LCA: life-cycle analysis
LPG: liquefied petroleum gas
MFSP: minimum fuel selling price
NPV: net present value
NREL: National Renewable Energy Laboratory
OPEX: operating expenses
SAF: sustainable aviation fuel
SBTi: Science-Based Targets initiative
TEA: techno-economic analysis.
