# Supplementary material for: Opportunity Assessment for Sustainable Aviation Fuel Production from Woody Biomass via Ex Situ Catalytic Fast Pyrolysis and Refinery Hydroprocessing
Source: Energy Fuels. 2025 Nov 5;39(45):21928–36. doi: 10.1021/acs.energyfuels.5c03405 (PMC12621182; doi:10.1021/acs.energyfuels.5c03405)
Supplement: Supplementary file 1 [file ef5c03405_si_001.pdf]

Supporting Information

Opportunity assessment for sustainable aviation fuel production from woody biomass via ex-situ catalytic fast pyrolysis and hydroprocessing

Nicholas A. Carlson[1], Michael S. Talmadge[1], Michael B. Griffin[1], Abhijit Dutta[1], Kristiina Iisa[1]*

[1.] National Renewable Energy Laboratory, Golden, CO 80401, USA

* Nicholas.Carlson@nrel.gov

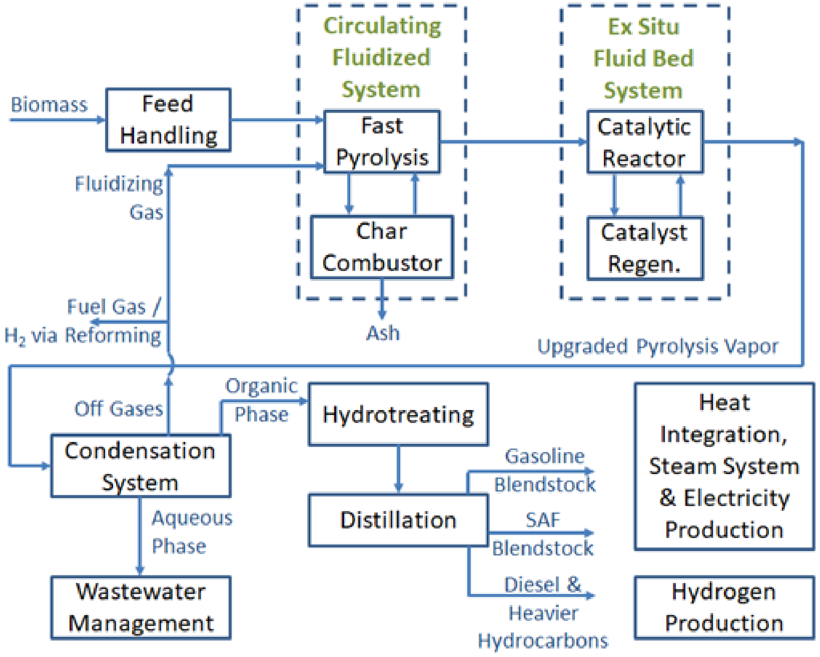

Figure S1. Simplified process flow diagram of commercial ex-situ catalytic fast pyrolysis process with hydroprocessing to produce drop-in transportation fuels.

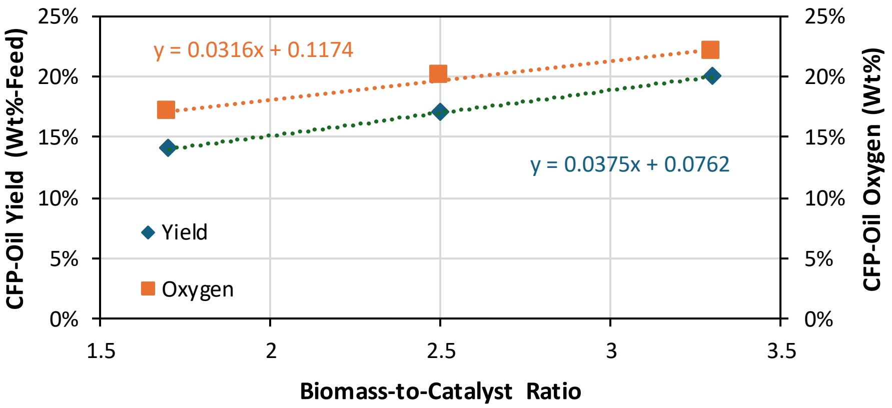

Figure S2. Catalytically upgraded (ex-situ fluidized bed reactor with ZSM-5 catalyst at 500 °C) pyrolysis oil yield and oxygen content as a function of biomass-to-catalyst ratio [1].

Table S1. Hydroprocessing yields and sustainable aviation fuel (SAF) cut properties across various reaction temperatures and CFP oil oxygen contents [1].

CFP-Oil Oxygen (Wt%)	Hydrotreating Temperature (°C)	Yields (Wt%)				SAF Properties		
		Gasoline	SAF	Diesel	Residue	Specific Gravity	Freeze Point (°C)	Final Boiling Point (°C)
17	300	13	45	15	25	0.95	-48	260
17	350	17	53	21	6	0.88	-80	271
17	385	26	50	17	4	0.85	-80	253
20	300	9	35	13	39	0.93	-80	253
20	385	26	46	21	3	0.84	-80	250
22	300	17	29	10	41	0.96	-53	276
22	385	22	47	23	4	0.85	-80	254

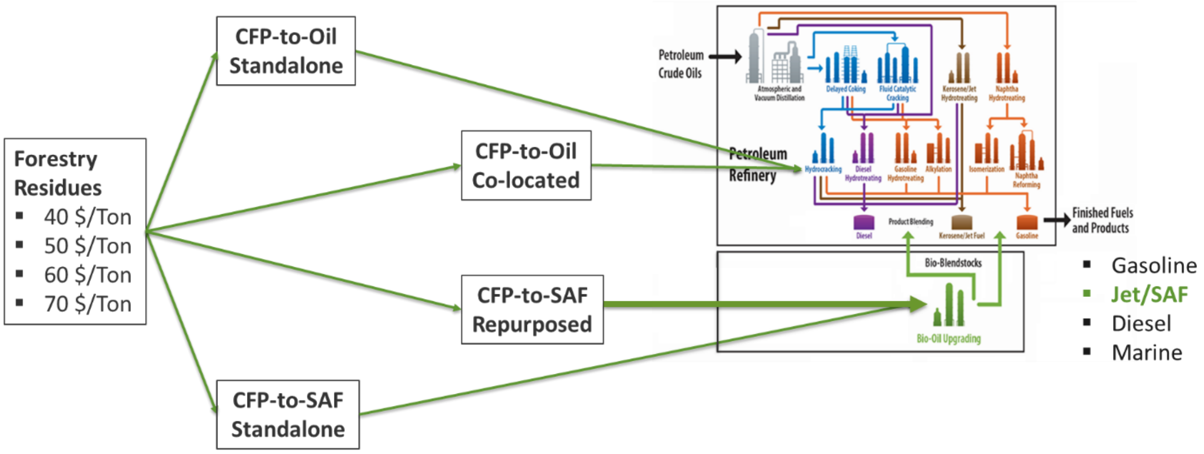

Figure S3. Graphical representation of CFP process configurations with standalone SAF and bio-oil production considered along with co-located options feeding a refinery hydrocracker. Repurposing and co-processing strategies were allowed for the refinery hydrocracker.

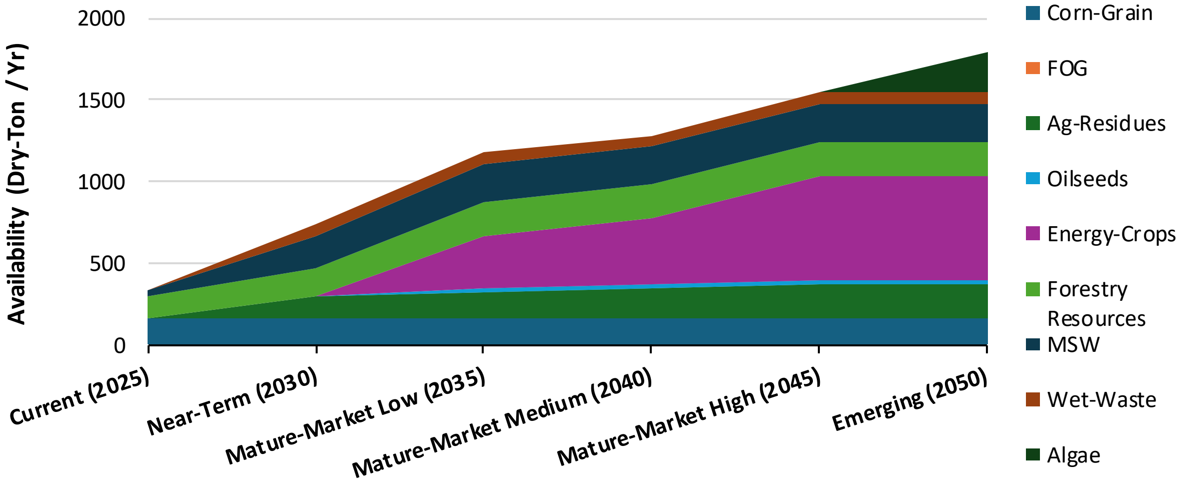

Figure S4. Biomass resource availabilities from the 2023 Billion Ton Study which can be sustainably collected and converted into biofuels trended over six scenarios which were aligned with years 2025 to 2050 for the purposes of this study [2].

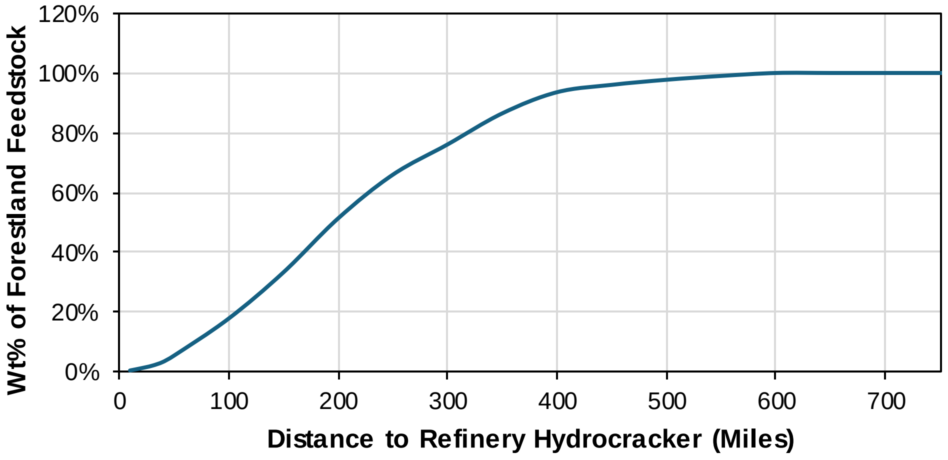

Figure S5. Weight percentage of U.S. forestland feedstock availability from the mature market high scenario in the 2023 Billion Ton Study as a function of distance to a U.S. refinery hydrocracker as reported in the Energy Information Administration's 2023 Refinery Capacity Report [2,3].

Table S2. Caption. ASTM Jet A fuel specifications reported along with fossil and CFP-to-SAF kerosene blendstock property assumptions. Fossil jet blendstock availabilities calculated using the EIA's refinery capacity report are also shown [3,4].

Property	ASTM Specification		Straight Run Kerosene	Hydrotreated Kerosene	Hydrocracked Kerosene	CFP-to-SAF Kerosene
	Value	Type				
**Distillation (% Evap @400 °F)**	10	Min	0	12	11	53
**Aromatics (Vol%)**	24	Max	21.48	18	21.73	~0
**Sulfur (Wt%)**	0.05	Max	0.20	0.01	0.01	~0
**Specific Gravity**	0.755 - 0.840	Range	0.829	0.809	0.807	0.843
**Luminometer #**	40	Min	44	46	41	†
**Smoke Point (mm)**	20	Min	19	26	24	†
**Freeze Point (°C)**	-40	Max	-27	-57	-50	-80
**U.S. Jet Blendstock Availability (B-Gal/Yr)**			16.8*	23.7	6.0	-

*SR-kerosene availability calculated using atmospheric crude distillation multiplied by mean kerosene yield minus kerosene hydrotreater capacity because SR-kerosene feeds that unit.
†Assumed equal to specification value in absence of analytical data.

References

(1) Griffin, M. B.; Iisa, K.; Dutta, A.; Chen, X.; Wrasman, C.; Mukarakate, C.; Yung, M. M.; Nimlos, M. R.; Tuxworth, L.; Baucherel, X.; Rowland, S. M.; Habas, S. E. Opening Pathways for the Conversion of Woody Biomass into Sustainable Aviation Fuel via Catalytic Fast Pyrolysis and Hydrotreating. *Green Chem.* **2024**, *26* (18), 9768–9781. https://doi.org/10.1039/D4GC03333G.

(2) M. H. Langholtz (Lead). *2023 Billion-Ton Report: An Assessment of U.S. Renewable Carbon Resources*; ORNL/SPR-2024/3103; Oak Ridge National Laboratory: Oak Ridge, TN, 2024.

(3) *Refinery Capacity Report*; U.S. Energy Information Administration (EIA): Washington, DC 20585, 2024. https://www.eia.gov/petroleum/refinerycapacity/index.php (accessed 2024-06-25).

(4) *ExxonMobil Jet A-1 Specifications*. https://www.exxonmobil.com/en/aviation/products-and-services/products/exxonmobil-jet-a-1 (accessed 2024-08-06).
